# High nutrient-use efficiency during early seedling growth in diverse *Grevillea* species (Proteaceae)

**DOI:** 10.1038/srep17132

**Published:** 2015-11-26

**Authors:** Tianhua He, William M. Fowler, Casey L. Causley

**Affiliations:** 1 Department of Environment and Agriculture, Curtin University, PO Box U1987, Perth, WA 6845, Australia

## Abstract

Several hypotheses have been proposed to explain the rich floristic diversity in regions characterised by nutrient-impoverished soils; however, none of these hypotheses have been able to explain the rapid diversification over a relatively short evolutionary time period of *Grevillea*, an Australian plant genus with 452 recognised species/subspecies and only 11 million years of evolutionary history. Here, we hypothesise that the apparent evolutionary success of *Grevillea* might have been triggered by the highly efficient use of key nutrients. The nutrient content in the seeds and nutrient-use efficiency during early seedling growth of 12 species of *Grevillea* were compared with those of 24 species of *Hakea*, a closely related genus. Compared with *Hakea*, the *Grevillea* species achieved similar growth rates (root and shoot length) during the early stages of seedling growth but contained only approximately half of the seed nutrient content. We conclude that the high nutrient-use efficiency observed in *Grevillea* might have provided a selective advantage in nutrient-poor ecosystems during evolution and that this property likely contributed to the evolutionary success in *Grevillea*.

Mediterranean climate regions, such as those in southwest Western Australia (SWA), the South African Cape region, and the Mediterranean Basin, have particularly diverse species and endemic rich flora and are considered globally significant^1^,^2^. Numerous hypotheses have been proposed to explain the floristic diversity of these biodiversity hotspots^3^. For example, Hopper proposed that landscape age contributed to the high diversity in SWA^4^, and Lengyel *et al*. reported higher global diversification rates in myrmecochorous lineages relative to their sister groups and speculated that myrmecochory contributed to the high diversification of SWA flora^5^. However, none of the current hypotheses can explain the extreme diversity of many species-rich genera in Australia, such as *Grevillea* in the family Proteaceae.

*Grevillea* is a predominantly Australian genus containing 362 currently recognised species or 452 taxa, which includes subspecies, with five species present in New Guinea, New Caledonia, and Sulawesi^6^. The evolutionary time period for species accumulation has emerged as a significant explanatory variable for biodiversity^3^,^4^, and this explanation is supported by fossil evidence^7^. *Grevillea* is one of the youngest genera, with only *c*. 11.2 million years of evolutionary history^8^, and it is the most species-rich genus in the Proteaceae family; thus, the greater-time-for-speciation theory does not explain the high numbers of species in this genus. However, Hopper proposed that taxa in old and climate-buffered landscapes, including *Grevillea* and other genera, such as the closely related genus *Hakea*, typically have a low extinction rate^4^. *Grevillea* species are generally dispersed by ants, which has been hypothesised to be a key evolutionary novelty that may account for the high diversification rate in this genus^5^. However, many other plant lineages in SWA, typically species that co-occur with *Grevillea* and are also dispersed by ants, have markedly fewer species within their genera. For example, ant-dispersed *Daviesia* (Fabaceae) diversified into 127 species over *c*. 26 million years, whereas ant-dispersed *Bossiaea* (Fabaceae) diversified into 80 species within *c*. 10 million years of evolutionary history^3^. Therefore, myrmecochory alone is unlikely to contribute to the high diversification rate in *Grevillea*. With a relatively short evolutionary history of 11.2 million years and as the third largest plant genus in Australia after *Acacia* and *Eucalyptus*, *Grevillea* probably has one of the highest net speciation rates of all large Australian plant clades.

Species in the family Proteaceae are highly adapted to and became diversified on the most nutrient-impoverished soils observed worldwide. Highly infertile soils have long been demonstrated to influence biological and physiological responses and speciation patterns^4^,^9^. Species have developed diverse morphological and physiological adaptations to limited nutrient availability in order to successfully establish in nutrient-limited soils^10^,^11^,^12^,^13^. These species typically exhibit highly efficient photosynthetic phosphorus use^14^.

In southwestern Australia, seed germination and successful seedling establishment predominantly occurs following winter rains after summer fires. Seed-stored nutrients, particularly nitrogen (N) and phosphorus (P), are crucial for the emergence and early growth of seedlings, and the efficient use of these nutrients is critical for the successful establishment of seedlings in severely nutrient-poor and water-limited environments. Nutrient limitation has recently been proposed as a potential driver for variation in species diversity^15^. Lambers *et al*. suggested that high plant species diversity on infertile soils, particularly in species-rich biomes, is associated with functional diversity for nutritional strategies, including nutrient-use efficiency^16^. Despite extensive research on plant species richness in relation to nutrient availability, few studies have attempted to explore the relationship between nutrient-use efficiency and speciation patterns. Here, we compare the nutrient concentration and content in seeds and nutrient-use efficiency during early seedling growth of *Grevillea* with those of the closely related genus *Hakea*, another Australian genus with 149 species that efficiently use seed nutrients during early seedling growth^16^. Our study aimed to provide unique insights into the potential mechanisms underlying the evolutionary success (i.e., high diversification rate) of *Grevillea*.

## Results

**Figure 1:**
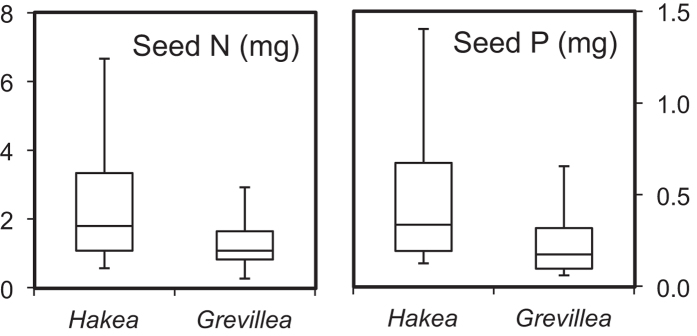
Comparison of nitrogen (N) and phosphorus (P) content per seed of the test species of *Hakea* and *Grevillea*. The middle lines in the box represent the median values, and the bottom and top of the box are the first and third quartiles. The ends of the whiskers are 1.5 interquartile range (IQR) above the third quartile and 1.5 IQR below the first quartile.

Seed mass varies markedly in both *Grevillea* (2.8–244 mg, 116 species/subspecies surveyed) and *Hakea* (2.6–501 mg, 153 species/subspecies surveyed), and *Grevillea* has a slightly higher median seed mass than *Hakea* (24.2 vs. 18.7 mg). The average seed masses of the 30 *Grevillea* species and 29 *Hakea* species used in the present study were statistically similar (Table 1). Nutrient assays on the 30 *Grevillea* species and 29 *Hakea* species revealed significantly higher concentrations of N and P in the *Hakea* seeds than in the *Grevillea* seeds (Table 1; Table S1). The total N and P contents per seed in *Hakea* were also much higher than in *Grevillea* (N: 2.66 vs. 0.56 mg, *P* = 0.002; P: 0.47 vs. 0.17 mg, *P* = 0.008; Fig. 1).

The seeds of the 25 *Hakea* species that were tested readily germinated without requiring pre-treatment, and they had an overall germination rate of 82%. *Grevillea* seeds (29 species) pre-treated with smoke water exhibited a significantly lower germination rate of 32% (Table 1). However, for the 24 *Hakea* and 12 *Grevillea* species that yielded a sufficient number of germinants for growth comparisons, the time period to expand the first true leaves was similar, although *Grevillea* plants had significantly fewer leaves after growing for 100 days. Both *Hakea* and *Grevillea* diverted more resources to the roots than to the shoots, with a statistically similar root:shoot ratio. *Grevillea* and *Hakea* species accumulated similar amounts of dry biomass and had similar root depths in the soil after 100 days of growth (Table 1).

**Figure 2:**
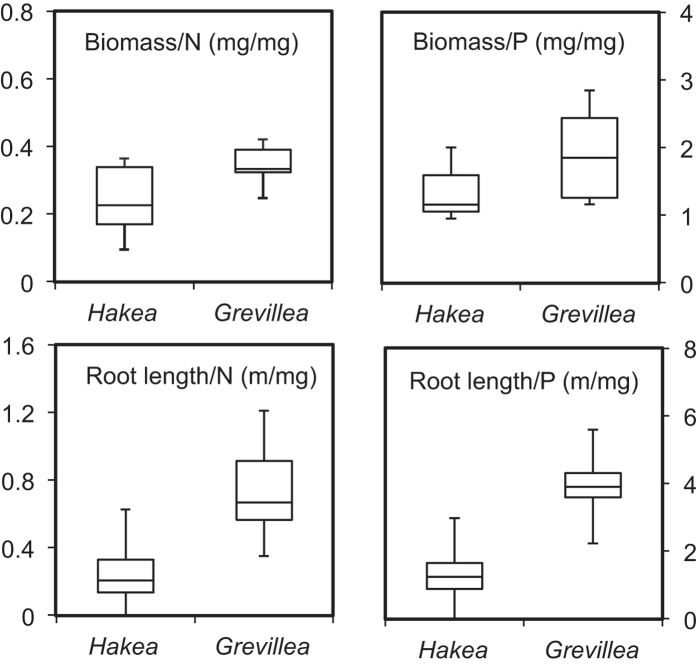
The nutrient-use efficiency (N and P) of the test species of *Hakea* and *Grevillea*. The middle lines in the box represent the median values, and the bottom and top of the box are the first and third quartiles. The ends of the whiskers are 1.5 interquartile range (IQR) above the third quartile and 1.5 IQR below the first quartile.

Further analysis of the seed nutrient content and seedling growth rate revealed that *Grevillea* species had significantly higher nutrient-use efficiencies (Table 1; Fig. 2). Compared with *Hakea*, for each unit of P in the seed, *Grevillea* accumulated 1.6-fold more dry biomass and had roots that descended 2.9 times deeper into the soil, whereas for each unit of N in the seed, *Grevillea* accumulated 1.4-fold more dry biomass and had taproots that descended 3.1 times deeper into the soil (Table 1, Fig. 2).

## Discussion

The seed nutrient concentration in *Grevillea* reported in the present study, particularly with respect to P, was only half of that reported in previous scattered studies. For example, Hocking reported an average of 11.4 ± 2.6 mg.g^−1^ P for ten *Grevillea* species^17^. The results of the present study on 30 species reported an average of 6.3 ± 2.3 mg.g^−1^. This discrepancy is unlikely to be a consequence of methodological errors in our analysis because the 29 *Hakea* species were assayed in the same batch in the present study, and they yielded an average P content of 13.6 ± 3.8 mg.g^−1^, which is similar to the 15.1 ± 4.2 mg.g^−1^ P concentration reported for 13 species from ten previous studies (except *Hakea pycnoneura*, which was reported to have an unusually high P concentration of 36 mg.g^−1^)^18^. Interestingly, although the N and P concentrations in the seeds were lower in *Grevillea* than in *Hakea*, the N:P ratios in both taxa groups were similar. Notably, the lower seed N and P concentrations in *Grevillea* relative to those in *Hakea* did not reflect dilution by seed mass because the tested species in the two groups had similar seed masses.

With only half the seed nutrient content compared with species from the closely related genus *Hakea*, the species in *Grevillea* achieved similar levels of seedling growth during the early stages. Indeed, both groups of plants expanded their first true leaves within a similar time, accumulated similar amounts of dry biomass, and diverted similar proportions of resources to the shoots and roots. Consequently, the results of this analysis suggest that compared with *Hakea*, *Grevillea* species use nutrients significantly more efficiently than do *Hakea*. This efficiency represents a clear ecological advantage over *Hakea* because *Grevillea* species can accumulate twice as much dry biomass and descend their roots three times deeper into the soil for every unit of nutrient in their seeds.

Seed-stored nutrients (N and P) are crucial for the successful establishment of seedlings in nutrient-impoverished landscapes. The rapid descent of the taproots to reach more reliable soil moisture reserves prior to the onset of summer droughts is critical for the survival of seedlings, particularly in Mediterranean climates with dry summer conditions^19^. Using fewer nutrients, *Grevillea* species can grow roots equal in length to those of *Hakea* and survive dry Mediterranean-type summers equally as well, and these characteristics provide a selective advantage to *Grevillea* compared with other organisms that might require additional nutrients in nutrient-impoverished soils.

Seed production is inversely proportional to the per-seed reserve of nutrients, particularly P, in nutrient-impoverished soils^20^. In the fire-prone environments of SWA, which are generally characterised by poor soil, resprouters (populations that predominantly regenerate through sprouts from the trunk or underground organs) typically produce fewer seeds than nonsprouters (populations that predominantly regenerate through seeds)^21^. However, no significant differences were observed in the P content between nonsprouting and resprouting species growing on P-impoverished soil in a survey of 41 species in SWA^18^. The lower nutrient requirements for seedling growth in *Grevillea* relative to *Hakea*, which has much higher nutrient (N and P) concentrations, might allow *Grevillea* species to produce more seeds regardless of the post-fire regeneration mode. Because of challenges in gauging the size of the soil seedbank, systematic surveys of *Grevillea* seed production are rare, although Pickup *et al*. reported a large seedbank for *Grevillea rivularis* at 193 ± 73 seeds m^–2^ 22. Because of its capacity to grow to 2.5 m tall and 3 m wide, this plant species has the potential to produce up to 2000 seeds per plant, which is much higher than that of most *Hakea* species^23^. Levin^24^ proposed that propagule-rich lineages are likely to have high speciation rates because the increased number of seeds increases the opportunities for ecological and geographical speciation, and this hypothesis could also be true for *Grevillea*. Interestingly, high propagule pressure has also been proposed as one of the mechanisms facilitating successful invasion^25^.

Species in the Proteaceae family grow well in the world’s most nutrient-impoverished landscapes, particularly P-impoverished soils. Apart from diverse and efficient nutrient-acquisition strategies^26^, high nutrient-use efficiency is likely another key adaptation. Recent studies have observed high photosynthetic P-use efficiency in SWA Proteaceae species^10^. Lambers *et al*. reported that Proteaceae species such as *Hakea* species (*Grevillea* species were not included) from severely P-impoverished soils extensively replace phospholipids with galactolipids and sulpholipids during leaf development to achieve a high photosynthetic P-use efficiency, and proposed the “sulphur-for-phosphorus” hypothesis^16^. Sulpice *et al*.^27^ observed that Proteaceae species grow with very low levels of ribosomes, particularly plastidic ribosomes, at early stages of leaf development. Many of the above studies involved *Hakea* species, although they rarely included *Grevillea* species; thus, additional research is required to test whether *Grevillea* have developed peak nutrient-use efficiency.

It can be speculated that once *Grevillea* overcome the barriers imposed by the two most limiting factors to its development, low soil nutrient availability and periodic water availability, these plants can invade most habitats. Indeed, species of *Grevillea* have been observed throughout Australia. High nutrient-use efficiency has interacted with multiple factors, including myrmecochory, a predominantly sexual reproduction strategy, and a high seed output, to allow the *Grevillea* genus to diversify into a group with 452 species/subspecies within only 11 million years.

## Methods

The seeds of *Grevillea* and *Hakea* used for the nutrient analyses and germination and seedling growth comparisons were acquired from Nindethana Seed Service (Albany, Western Australia, Australia). Genus-wide seed mass information was extracted from the Seed Information Database^28^. The nutrient analysis was conducted on seeds from 59 species (29 *Hakea* and 30 *Grevillea*, Table S1). These species cover the major intra-generic taxonomy sections and provenances; therefore, they represent a wide diversity of the respective genera. For each species, up to 20 g of dry seeds were analysed for N and P concentrations at the ChemCentre (Perth, Western Australia, Australia) using the combustion method for N and inductively coupled plasma spectrometry for P.

A total of 52 species (25 *Hakea* and 27 *Grevillea* species, Table S1) were used in the germination trials. Each species had three replicates, and there were up to 30 seeds per replicate (depending on seed size). Each replicate was contained within a 120-mm sterile Petri dish with 2 layers of sterilized Whatman Grade 1 filter paper and sealed with Parafilm. Petri dishes with *Hakea* seeds were filled with 5 mL autoclaved deionised water, whereas those with *Grevillea* seeds (because of inherent dormancy) were filled with 5 mL 10% smoke water solution (Regen 2000 Smokemaster); previous studies have suggested that *Grevillea* seed germination is enhanced by smoke stimulation^29^. To reduce the risk of fungal growth throughout the germination experiment, the smoke water was passed through a 0.20-μm filter. Germination was conducted in an environmental chamber at a constant 15 °C with a 12 hour light/dark cycle. The germinants (seeds with radicle emergence ≥1 mm) were counted and recorded every two days for a period of up to 60 days. Seeds that did not germinate were assessed for viability using a cut test to visually assess the health of the embryo. The Petri dishes were randomised within the environmental chamber in each experiment.

For each of the species germinated in the above-described germination phase (12 *Grevillea* and 24 *Hakea* species, with no or minimal germination recorded in some species), ten germinants (or fewer when available) were individually planted in PVC tubes (100 cm in length and 5 cm in diameter) with a substrate of washed white sand (with no nutrients) and placed in a hoop house. The seedlings were watered (20 mL) every 2 to 3 days and grown for 100 days (from late winter to spring 2014) prior to harvesting. The measurements taken at harvest were leaf number, shoot length, root length and fresh and dry shoot and root mass. The dry biomass of the shoot and root was recorded after the samples were oven dried at 70 °C for 48 hours.

The nutrient-use efficiency (N and P) was defined as the amount of biomass produced or root length gained per unit of nutrient (mg) in the seeds. The nutrient-use efficiencies of *Hakea* and *Grevillea* were compared. Comparisons of the parameter values between *Grevillea* and *Hakea* were analysed with a t-test and implemented in PAST^30^. *P* < 0.05 was considered to represent statistical significance.

## Additional Information

**How to cite this article**: He, T. *et al*. High nutrient-use efficiency during early seedling growth in diverse *Grevillea* species (Proteaceae). *Sci. Rep*. **5**, 17132; doi: 10.1038/srep17132 (2015).

## Supplementary Material

Supplementary Information

## Figures and Tables

**Table 1:** Seed mass and nutrient concentration (N and P), seed generation, and seedling growth in *Hakea* and *Grevillea*.

	*Hakea*	*Grevillea*	t-test, P value
Number of species	149^a^	362^b^	–
Age (million years)^c^	15.8	11	–
Seed mass (mg)^d^	30.4 (2.6–501.0)	34.9 (2.8–244.0)	–
Seed mass (mg)^e^	33.3 ± 29.2	39.5 ± 15.5	t = −0.40, P = 0.703
Seed N (%)	7.61 ± 1.36	3.53 ± 0.79	t = −1.09, P < 0.001
Seed P (%)	1.36 ± 0.38	0.63 ± 0.23	t = −1.48, P < 0.001
Germination rate	0.82 ± 0.12	0.32 ± 0.15	t = −5.07, P < 0.001
Days to first true leaf	32 ± 6	31 ± 5	t = 0.02, P = 0.942
Number of leaves	16.7 ± 5.4	8.3 ± 1.2	t = −3.57, P = 0.002
Root/shoot biomass	1.67 ± 0.71	1.34 ± 0.55	t = −1.27, P = 0.218
Total dry biomass (g)	0.54 ± 0.39	0.28 ± 0.13	t = −1.09, P = 0.093
Root length (mm)	468 ± 198	580 ± 181	t = −1.48, P = 0.152

^a^6;

^b^6;

^c^8;

^d^28;

^e^:species examined in this study.
