# Supplementary material for: High nutrient-use efficiency during early seedling growth in diverse Grevillea species (Proteaceae)
Source: Sci Rep. 2015 Nov 26;5:17132. doi: 10.1038/srep17132 (PMC4660852; doi:10.1038/srep17132)
Supplement: Supplementary Information [file srep17132-s1.doc]

**High nutrient-use efficiency during early seedling growth in diverse *Grevillea* species (Proteaceae)**

Tianhua He, William M. Fowler and Casey L. Causley

Table S1 Seed mass, nutrient concentration and content (nitrogen and phosphorus) in 30 *Grevillea* and 29 *Hakea species*

*Grevillea*

|  |  | Concentration | | Content (per seed) | |
| --- | --- | --- | --- | --- | --- |
| Species | Seed mass (mg) | Nitrogen (mg g^-1^) | Phosphorus (mg g^-1^) | Nitrogen (mg) | Phosphorus (mg) |
| *Grevillea aurea* | 33.3 | 35.1 | 4.2 | 1.169 | 0.14 |
| *Grevillea baileyana* | 13.6 | 38.4 | 6.3 | 0.522 | 0.086 |
| *Grevillea banksii* | 70.5 | 31.2 | 4.9 | 2.2 | 0.345 |
| *Grevillea candelabroides* | 10.6 | 28.0 | 8.6 | 0.297 | 0.091 |
| *Grevillea decora* | 37.7 | 34.4 | 4.3 | 1.297 | 0.162 |
| *Grevillea decurrens* | 61.7 | 43.0 | 5.5 | 2.653 | 0.339 |
| *Grevillea eriobotrya* | 137.8 | 16.0 | 5.6 | 2.205 | 0.772 |
| *Grevillea eriostachya* | 26.4 | 32.0 | 7.6 | 0.845 | 0.201 |
| *Grevillea excelsior* | 47.5 | 35.6 | 10.2 | 1.691 | 0.485 |
| *Grevillea johnsonii* | 52.9 | 20.9 | 3.5 | 1.106 | 0.185 |
| *Grevillea leucopteris* | 89.7 | 25.9 | 9.3 | 2.323 | 0.834 |
| *Grevillea mimosoides* | 36.1 | 59.1 | 5.9 | 2.134 | 0.213 |
| *Grevillea monticola* | 41.5 | 38.7 | 3.7 | 1.606 | 0.154 |
| *Grevillea paniculata* | 15.6 | 31.7 | 5.2 | 0.495 | 0.081 |
| *Grevillea petrophiloides* | 10.7 | 38.6 | 6.8 | 0.413 | 0.073 |
| *Grevillea plurijuga* | 31.1 | 40.0 | 9.8 | 1.244 | 0.305 |
| *Grevillea polybotrya* | 17.3 | 38.5 | 10.6 | 0.666 | 0.183 |
| *Grevillea pteridifolia* | 34.1 | 37.2 | 5.3 | 1.269 | 0.181 |
| *Grevillea pterosperma* | 31.2 | 29.8 | 10.6 | 0.93 | 0.331 |
| *Grevillea pulchella* | 30.9 | 35.5 | 3.3 | 1.097 | 0.102 |
| *Grevillea refracta* | 103.5 | 41.9 | 5.7 | 4.337 | 0.59 |
| *Grevillea robusta* | 10.3 | 41.4 | 6.5 | 0.426 | 0.067 |
| *Grevillea stenobotrya* | 28.9 | 28.8 | 5.8 | 0.832 | 0.168 |
| *Grevillea synapheae* | 29.1 | 34.8 | 3.5 | 1.013 | 0.102 |
| *Grevillea teretifolia* | 9.2 | 30.9 | 6.9 | 0.284 | 0.063 |
| *Grevillea tetragonoloba* | 24.9 | 41.1 | 6.7 | 1.023 | 0.167 |
| *Grevillea triloba* | 23.1 | 40.0 | 4.1 | 0.924 | 0.095 |
| *Grevillea triternata* | 30.1 | 29.2 | 4.2 | 0.879 | 0.126 |
| *Grevillea vestita* | 34.6 | 39.9 | 5.9 | 1.381 | 0.204 |
| *Grevillea wilsonii* | 46.5 | 42.5 | 8.1 | 1.976 | 0.377 |

*Hakea*

|  |  | Concentration | | Content (per seed) | |
| --- | --- | --- | --- | --- | --- |
| Species | Seed mass (mg) | Nitrogen (mg g^-1^) | Phosphorus (mg g^-1^) | Nitrogen (mg) | Phosphorus (mg) |
| *Hakea adnata* | 23.8 | 79.4 | 17.8 | 1.890 | 0.424 |
| *Hakea arborescens* | 107.5 | 76.0 | 10.0 | 8.170 | 1.075 |
| *Hakea bucculenta* | 15.3 | 52.6 | 16.9 | 0.805 | 0.259 |
| *Hakea carinata* | 11.3 | 79.0 | 11.1 | 0.893 | 0.125 |
| *Hakea corymbosa* | 11.4 | 87.6 | 14.5 | 0.999 | 0.165 |
| *Hakea cycloptera* | 64.5 | 60.1 | 13.2 | 3.876 | 0.851 |
| *Hakea denticulata* | 27.3 | 96.1 | 9.2 | 2.624 | 0.251 |
| *Hakea drupacea* | 14.5 | 77.0 | 19.1 | 1.117 | 0.277 |
| *Hakea epiglottis* | 13.1 | 90.0 | 16.8 | 1.179 | 0.221 |
| *Hakea gibbosa* | 71.8 | 67.6 | 11.4 | 4.854 | 0.819 |
| *Hakea hookeriana* | 78.4 | 81.3 | 17.1 | 6.374 | 1.341 |
| *Hakea laurina* | 20.1 | 69.3 | 11.3 | 1.393 | 0.227 |
| *Hakea leucoptera* | 30.7 | 58.2 | 11.1 | 1.787 | 0.341 |
| *Hakea lissosperma* | 13.2 | 74.2 | 14.6 | 0.979 | 0.193 |
| *Hakea lorea* | 55.8 | 59.6 | 7.1 | 3.326 | 0.396 |
| *Hakea megalosperma* | 158.3 | 88.6 | 12.2 | 14.025 | 1.931 |
| *Hakea microcarpa* | 13.2 | 81.4 | 9.7 | 1.075 | 0.128 |
| *Hakea multilineata* | 11.3 | 51.3 | 14.5 | 0.580 | 0.164 |
| *Hakea ochroptera* | 33.9 | 64.5 | 11.2 | 2.187 | 0.380 |
| *Hakea oleifolia* | 11.7 | 103 | 16.2 | 1.205 | 0.190 |
| *Hakea orthorrhyncha* | 32.9 | 60.8 | 21.6 | 2.000 | 0.711 |
| *Hakea platysperma* | 37.6 | 85.7 | 12.6 | 3.222 | 0.474 |
| *Hakea polyanthema* | 65.7 | 87.0 | 12.4 | 5.716 | 0.815 |
| *Hakea recurva* | 20.1 | 76.5 | 8.8 | 1.538 | 0.177 |
| *Hakea rostrata* | 19.4 | 87.5 | 23.1 | 1.698 | 0.448 |
| *Hakea rugosa* | 18.6 | 95.1 | 15.2 | 1.769 | 0.283 |
| *Hakea sericea* | 29.4 | 86.1 | 12.2 | 2.531 | 0.359 |
| *Hakea strumosa* | 62.2 | 62.6 | 10.9 | 3.894 | 0.678 |
| *Hakea vittata* | 13.2 | 78.7 | 13.4 | 1.039 | 0.177 |
